# Intergenerational Cycle of Obesity and Diabetes: How Can We Reduce the Burdens of These Conditions on the Health of Future Generations?

**DOI:** 10.1155/2011/596060

**Published:** 2011-10-29

**Authors:** Marie-Claude Battista, Marie-France Hivert, Karine Duval, Jean-Patrice Baillargeon

**Affiliations:** Division of Endocrinology, Department of Medicine, University of Sherbrooke, Sherbrooke, QC, Canada J1H 5N4

## Abstract

Prepregnancy overweight or obesity and excessive gestational weight gain have been associated with increased risk of maternal and neonatal complications. Moreover, offspring from obese women are more likely to develop obesity, diabetes mellitus, and cardiovascular diseases in their lifetime. Gestational diabetes mellitus (GDM) is one of the most common complications associated with obesity and appears to have a direct impact on the future metabolic health of the child. Fetal programming of metabolic function induced by obesity and GDM may have intergenerational effect and thus perpetuate the epidemic of cardiometabolic conditions. The present paper thus aims at discussing the impact of maternal obesity and GDM on the developmental programming of obesity and metabolic disorders in the offspring. The main interventions designed to reduce maternal obesity and GDM and their ability to break the vicious circle that perpetuates the transmission of obesity and metabolic conditions to the next generations are also addressed.

## 1. Introduction

Obesity is a major public health problem that was identified as an epidemic by the World Health Organization [1]. The US Centers for Disease Control and Prevention estimates that 34% of adults 20 years of age and over are obese (body mass index (BMI) above 30 kg/m^2^), in addition to another 34% who are classified as overweight (BMI 25–30 kg/m^2^) for a total of over two-thirds of Americans with inappropriately high body weight. Women are thus more likely to enter pregnancy overweight or obese nowadays. In fact, among a sample of 75.000 delivering women recruited all over USA, 20% were obese [2]. Moreover, offspring from obese women is more likely to develop obesity, diabetes mellitus, and cardiovascular diseases in their lifetime, often at a younger age [3]. This paper will thus focus on maternal obesity and gestational diabetes mellitus (GDM) as determinants for the developmental programming of obesity and metabolic disorders in the offspring. We are also presenting the main interventions tested to reduce those two important maternal burdens and how they can break the vicious circle that perpetuates the transmission of obesity and metabolic conditions to the next generations.

## 2. Gestational Weight and Metabolic Complications

### 2.1. Appropriate Gestational Weight Gain

Due in part to concerns about the increasing prevalence of obesity in reproductive-age women, the Institute of Medicine (IOM; which is a nonprofit American organization serving as adviser to the nation to improve health http://www.iom.edu/About-IOM.aspx) recently released new guidelines (Table 1) for gestational weight gain to better reflect the growing body of evidence in favour of lowering weight gain recommendations for overweight and obese pregnant women [4]. In comparison to the previous 1990 guidelines, 1 out of 6 women were differently classified using the new 2009 guidelines, that is, fewer pregnant women were classified as underweight, normal weight, or obese, and more as overweight [5]. Moreover, 17.1% of appropriate gainers based on 1990 recommendations would now be classified as overgainers [5]. As some people were worried by these severe guidelines, Einerson et al. showed, in a retrospective study, that the new recommended weight gain thresholds were safe for both the mothers and the developing child [6]. In fact, those new weight gain thresholds were clinically effective in reducing caesarean and pregnancy-induced hypertension, and no increase in the proportion of low-birth weight infants was recorded in 691 participants as compared to the proportion observed in obese women with appropriate weight gain based on 1990 recommendations [6].

However, according to these new guidelines, Weisman et al. reported from a population-based cohort study that 40% of normal weight women exceeded the IOM recommendations, while excess weight gain was observable in 63% and 62% of overweight and obese women, respectively, [7]. The same group also showed that prepregnancy overweight women were 3 times more likely to exceed the recommended weight gain than normal-weight women [7]. In a recent observational study including 144 30-week pregnant women, 33% of participants reported an excessive gestational weight gain [8]. Factors associated with excess weight gain included prepregnancy overweight as well as low physical activity and high-food intake during pregnancy, as reported by the participants. On the other hand, some factors seem to be associated with lower risk of excess weight gain such as menarche occurring at an older age and increased hours of sleep [8]. In summary, these results suggest that pregnant women frequently exceed the recommended gestational weight gain, but it may be feasible to optimize weight loss by promoting physical activity and healthy eating among women of reproductive age.

### 2.2. Obesity-Associated Obstetrical and Neonatal Complications

Prepregnancy overweight or obesity and excessive pregnancy weight gain both have been independently associated with increased risk of complications. Indeed, several studies demonstrated the association between increased maternal BMI and higher risks of obstetric and neonatal complications [9]. Obese women are at increased risk of complications over the whole spectrum of peripregnancy period: antepartum, intrapartum, intraoperative, postoperative, and postpartum (for review, see [10, 11]). Briefly, at the maternal level, obesity increases the risk of menstrual disorders [12], infertility [13], miscarriage [14], pregnancy-induced hypertension and preeclampsia [15], GDM [16], induction of labour and caesarean section [17], and haemorrhage, infection, and venous thromboembolism. At the level of the newborn, maternal obesity is also associated with many complications: macrosomia (defined as a birth weight >4.000 g), shoulder dystocia [18], fetal distress, and perinatal morbidity/mortality (for review, see [10]). Still birth [19] and birth defects such as hydrocephaly, omphalocele, heart, and neural tube defects [20, 21] are among the disastrous neonatal outcomes that have been associated to maternal obesity (identified as the top modifiable risk factor). In a large population-based retrospective cohort study [22] including singleton macrosomic live births infants, it was demonstrated that the overall risk of obstetrical complications was increased almost 3-fold in obese (BMI ≥ 30 kg/m^2^) as compared to nonobese mothers (BMI < 30 kg/m^2^) (17% versus 6%). Macrosomic neonates born from obese mothers were at increased risk of birth injury, hyaline membrane disease, meconium aspiration syndrome, and more required assisted ventilation. Project Viva study [23] analysed data from 2.012 recruited mother-child pairs in order to determine the level of gestational weight gain which was associated with the lowest predicted prevalence of adverse obstetrical and neonatal outcomes. They showed that the mean total gestational weight gain was 15.6 kg. The lowest predicted outcome prevalence occurred with a weight gain of 11.2 kg for women with prepregnancy BMI in the normal range, with a weight loss of 1.2 kg for overweight women, and with a weight loss of 7.6 kg for obese participants.

Weight gain between each pregnancy (or not returning to prepregnancy body weight) is also associated with increased adverse health issues. Indeed, a large population-based Swedish cohort study including women who had 2 consecutive live-birth pregnancies robustly showed that in comparison to women whose prepregnancy BMI did not change much between both pregnancies (−1 to 0.9 BMI points), mothers who gained more than 3 units of BMI between pregnancies increased by 30% to 110% their adjusted estimated risks of important complications (odds ratio of 1.78 for preeclampsia, 1.76 for gestational hypertension, 2.09 for GDM, 1.32 for caesarean delivery, 1.63 for stillbirth, and 1.87 for large-for-gestational-age birth). These risks were associated with the amount of weight change between pregnancies, and this association was also noted in women who had a healthy prepregnancy BMI for both pregnancies [24]. This example underscores the point that women do not necessarily need to be in transition from a normal BMI to overweight or obese BMI, but those relatively small increases in BMI were associated with adverse outcomes. Collectively, these data emphasize the importance of adequate gestational weight gain in pregnant obese women, as recently recommended by the Institute of Medicine, in order to avoid maternal and neonatal complications. 

## 3. Gestational Diabetes Mellitus (GDM)

### 3.1. Definition and Diagnosis

According to the International Association of Diabetes and Pregnancy Study Groups (IADPSG) [25], GDM is defined as an impaired glucose tolerance that is first recognized during pregnancy, and it occurs in approximately 7% of pregnancies in the USA. Based on a meta-analysis, GDM incidence varies from 1.3% to 19.9% depending on screening and diagnostic guidelines that are followed and the study populations [26]. In 2010, new recommendations brought up by the IADPSG were approved to diagnose GDM [27]. This guideline suggests to screen with a 75 g oral glucose tolerance test (OGTT) without prior glucose challenge and to diagnose GDM if the fasting plasma glucose is ≥5.1 mmol/L and/or the 1-hour postload plasma glucose is ≥10.0 mmol/L and/or the 2-hour postload plasma glucose is ≥8.5 mmol/L.

### 3.2. Pathophysiology of Gestational Diabetes

Normal pregnancy is characterized by an insulin-resistant state in order to fulfill the increasing metabolic demand ordered by the developing foetus. The physiologic result of insulin resistance is thus an increase in insulin secretion by the pancreatic *β*-cells in order to compensate for reduced insulin action. However, women who fail to do so will progressively develop GDM [28]. In GDM, insulin resistance and the relative insulin deficiency due to pancreatic *β*-cell dysfunction are the primary metabolic changes. As gluconeogenesis increases, as a result of hepatic insulin resistance, and relative insulin deficiency is exacerbated, hyperglycemia becomes more severe. However, many questions still remain in order to adequately understand the mechanisms by which GDM takes place. It is not in the scope of this paper to discuss the pathophysiology of GDM, but mechanisms presently under investigation include the role of genetic factors, glucose transport activity, adipokines defects, and adipose tissue dysregulation (for review, see [29]).

### 3.3. Relationship between Gestational Diabetes and Maternal Obesity

Importantly, overweight and obese women are more insulin resistant than their lean counterparts and also more prone to *β*-cell dysfunction [30], which is mainly due to adipose tissue dysregulation and largely influenced by ethnicity and age [31]. For these reasons, obesity is an important determinant of the long-term risk of developing type 2 diabetes in genetically predisposed individuals. Consequently, overweight or obese women begin their pregnancy with insulin resistance and increased predisposition for *β*-cell dysfunction, which could result in GDM with the pregnancy-related progression of insulin resistance. An excess of weight gain during pregnancy would further worsen these phenomena and increase the risk of GDM as well, even in women with normal prepregnancy weight.

GDM is thus one of the most common obstetrical complications associated with obesity. The Project Viva study explored the relationship of trimester-specific rate of weight gain with impaired glucose tolerance during pregnancy [32]. In this study, the authors showed that the median rate of weight gain during early pregnancy (less than 13 weeks) was of 0.22 kg/week, while it was of 0.50 kg/week during midpregnancy. Accordingly, they showed that women who gained more than 0.22 kg/week during early pregnancy increased by 40% their odds of developing impaired glucose tolerance even if they gained more than 0.50 kg/week during midpregnancy. Moreover, women who were high gainers during early and midpregnancy doubled their odds for impaired glucose tolerance diagnosed during 2nd trimester [32]. By analyzing data from 7 states using the Pregnancy Risk Assessment Monitoring System (PRAMS), Kim et al. showed that the proportion of GDM that was attributable to overweight, obesity and extreme obesity was, respectively, of 15.4%, 9.7%, and 21.1% [33]. The overall population-attributable fraction for overweight/obesity, after subtracting women with normal weight, was 46.2%. However, it was recently shown in 36.597 Canadian women, that the increased incidence of glucose disorders during pregnancy was not explained by higher maternal prepregnancy adiposity [34]. These contradictory results between studies may be explained by different population ethnic background and patterns of gestational weight gain, which are important factors to consider in the interpretation of studies on GDM.

### 3.4. Gestational Diabetes-Associated Neonatal Complications

Excessive fetal growth is probably the most frequent and important outcome of GDM. It was long known that the nutritional status of the mother and accordingly her glycemic control may directly be in relation with her infant growth. This concept was based on studies reporting that macrosomia occurs as a result of fetal insulin hypersecretion in response to the mother rise of glycemia [35]. On the other hand, it was shown in pregnant rats that maternal hypoglycemia generates intrauterine growth restriction of the offspring, which was accompanied by fetal hypoglycemia and hypoinsulinemia [36]. Accordingly, while the mother glucose can diffuse through the placental barrier [37], maternal insulin cannot [38]. So, in situation of mother hyperglycemia, the foetus is consequently exposed to important quantities of glucose. In order to regulate its own glucose homeostasis, the foetus increases its insulin secretion, which is an important growth factor in the developing foetus. The foetus is thus more exposed to insulin leading to excessive growth and later programming of metabolic functions [39]. Additionally to macrosomia, many of the perinatal complications that have been associated with obesity are also known to be associated with GDM [40]. These include increased risks for preeclampsia, caesarean, stillbirth, spontaneous abortion and congenital malformations.

The question thus arising is whether the increased risk of adverse perinatal outcome in obese women is related to obesity *per se* or the increased risk of developing GDM. The Hyperglycemia and Adverse Pregnancy Outcome (HAPO) study attempted to address such question. This trial enrolled more than 23.000 mothers and baby pairs. This study showed a strong linear association between fasting and postchallenge glucose and the incidence of macrosomia and neonatal adiposity [41]. They also clearly showed that the impact of hyperglycaemia on the foetus was present at a much lower glucose concentration than previously thought [42]. This study thus suggests that complications may arise even with gestational glucose levels below the thresholds of GDM. Furthermore, a retrospective study of 413 women with GDM or impaired glucose tolerance found that fetal macrosomia was not associated with maternal glucose values, except fasting glucose levels between 32 and 35 weeks of pregnancy, while maternal obesity was a strong risk factor for macrosomia throughout these pregnancies [43]. On the other hand, a recent study suggested that pre-GDM was a stronger predictor of increase in birth defect prevalence than maternal obesity in their study population [44]. So the question of whether adverse perinatal outcomes in obese pregnant women are due to GDM (clinical or subclinical) is still unanswered and remains an open field for research.

## 4. Fetal Programming of Adult Diseases

In addition to the previously cited neonatal complication induced by maternal obesity and GDM, offspring born from these women are more likely to develop health problems later in life. In fact, in the early 1990, David Barker published a paper in the British Medical Journal entitled *“The fetal and infants origin of adult disease. The womb may be more important than the home” *[45]. This concept of fetal programming or developmental plasticity states that an insult or stress, occurring during period of maximal growth and development of an organ, overcome normal physiological processes. In such situation, the organ needs to adapt to its new environment, which then conducts to adaptation/reprogramming of functions of this organ [46]. In his 1990 paper, Barker was visionary as he stated that “research should be redirected towards the intrauterine environment rather than the environment in later childhood-housing, family income, diet, and other influences” [45].

Of the first studies that were conducted to support the concept of fetal programming, maternal nutrition, either deficient or excessive, during pregnancy was pinpoint to have various and often deleterious effects on the offspring. Of the most critical and cited examples are the Dutch Famine and some studies conducted in Pima Indians. During World War II, food supply and calories intake were dramatically restricted in the Netherlands. This dark period was called the Dutch Hunger Winter. Consequently, foetuses that were exposed to this famine during mid- to late pregnancies had small placentas, were born small-for-gestational age when compared to those foetuses exposed in the early phase of pregnancy. As these foetuses became adults, the proportion of individuals suffering from glucose intolerance and insulin resistance was greater among those whose fetal insult occurred during mid- to late pregnancy. However, atherogenic lipid profile, increased BMI and raised cardiovascular risk were greater among those who were exposed during early pregnancy [47].

Pima Indians are a recognized population for increased prevalence of obesity and type 2 diabetes particularly since their nutritional income was greatly modified by a typical Westernized diet in the past few decades [48]. In comparison to infants born from mothers who developed diabetes after pregnancy, those born from women who had diabetes before pregnancy were more prone to obesity, had higher blood glucose and glycated haemoglobin levels, lower C-HDL concentration and were more likely to develop diabetes during their childhood [49, 50]. These results suggests that the period when the fetal insult occur is very determinant in the reprogramming of functions and that either a poor or a too rich maternal nutrition can lead to a U-shaped association for risk of future adult diseases, with both low and high birth weight being associated with risk of adverse health consequences.

## 5. Long-Term Health Effect of Gestational Obesity on the Offspring

### 5.1. Clinical Studies

Major factors associated with fetal adiposity are maternal prepregnancy BMI and weight gain during pregnancy [51]. Many studies have reported that mother prepregnancy BMI and gestational weight gain were positively associated with offspring BMI at birth as well as during infancy, childhood and early adulthood [52–56]. A recent retrospective study of 8.400 children in the US found that children born from obese mothers were twice as likely to be obese at 24 months of age [57]. It was shown that at time of birth, infants born from obese women had increased adiposity, homeostasis model assessment of insulin resistance (HOMA-IR, a fasting index estimating insulin resistance), cord blood leptin and interleukin-6 (IL-6) concentrations. Moreover, fetal adiposity and mother prepregnancy BMI were both highly correlated with fetal HOMA-IR [58].

In a recent prospective cohort of 146.894 offspring from 136.050 families, Lawlor and colleagues explored whether and how maternal weight gain is associated with increased offspring BMI at 18 years old. They showed that maternal weight was positively associated to young adult adiposity. They also provided evidences that offspring adiposity later in life was mostly explained by familial characteristics in those born to normal weight mothers, but mainly influenced by intrauterine mechanisms in overweight and obese mothers [59]. Moreover, as caesarean is one important adverse outcome associated with maternal obesity, it was shown that offspring born by caesarean were more likely to suffer from obesity at age 23–25 years in comparison to those born vaginally, even after appropriate adjustments (15% versus 10%) [60]. In the longitudinal 1986 Northern Finland Birth Cohort study including 4.168 participants, Pirkola et al. found that infants born from overweight and GDM mothers had a 40% and 26% higher prevalence for being either overweight or having abdominal obesity at the age of 16 years. This risk was also present in the subgroup of infants born to overweight mothers who were free of GDM during pregnancy [61]. This significant impact of maternal obesity on child obesity at a later age, independent of maternal glycemic status, was confirmed in other studies [62]. This led us to speculate that maternal obesity, either directly or through its associated complications, is a risk factor for increased adiposity and reprogramming of metabolic functions in the offspring at birth as well as the perpetuation of this increased adiposity into adulthood.

### 5.2. Potential Mechanisms for the Long-Term Impacts of Gestational Obesity on the Offspring

#### 5.2.1. Metabolic and Hormonal Theories

In order to better understand the fetal programming of increased fat mass observed in human epidemiological studies, animal model of maternal obesity was developed. Although not yet well understood, few mechanisms of action for such long-lasting effect in offspring were proposed. The most common animal model is the one using a high-fat diet either during gestation alone or in combination to lactation period. However, one question that can be addressed for the validity of this model in term of similarity with real obesity observed in women is whether maternal obesity acts as a programming agent *per se* or whether other aspects of the obesity-inducing diet drive the fetal responses. To answer this question, Spraque-Dawley rats where fed an obesogenic liquid diet by intragastric cannulation to induce obesity in those dams [63]. At mating period, dams were transferred to a control diet and thereafter offered back their obesogenic diet. In order to specifically target the intrauterine life as an obesogenic environment, after birth, pups born from obese animals were cross-fostered to lean dams at birth. In comparison with those born from lean dams, the offspring of obese dams exhibited increased adiposity and insulin resistance until postnatal day 130. This study therefore strongly suggests an independent effect of maternal obesity, specifically during the fetal period, on risk of obesity in the offspring. In line with this study, Bayol et al. showed in 10-week-old rats born to mothers fed a westernized high-fat diet throughout the course of gestation and lactation an exacerbated preference for palatable foods, at the expense of protein-rich foods, that was accompanied by increased body weight and fat mass when compared to control offspring [64]. In these rats, exacerbated adiposity was accompanied by elevated insulin, glucose, triglycerides and cholesterol plasma concentrations. Furthermore, a group of offspring from mothers fed the westernized diet during pregnancy and lactation were switched to a balanced chow diet after weaning. In comparison to offspring born from dams who were never exposed to prenatal junk food diet, exposed offspring exhibited a relative increase in perirenal fat pad mass (a visceral fat depot) and adipocyte hypertrophy, which is believed to reflect reduced ability for adipogenesis and hyperplasia, as well as modulation of genes involved in glucose and insulin regulation and in adipose tissue functions [65]. These studies demonstrate that an obesogenic maternal diet during pregnancy and lactation may directly contribute to the development of total body adiposity, adipose tissue dysfunction, central fat accumulation and metabolic disease later in life.

Obesity, insulin resistance, glucose intolerance and metabolic syndrome were also shown after culling pups to smaller litter size during lactation (F0 generation) [66]. Generational transmission of these pathological states, except for obesity, was observed in the aging offspring born from rats of the F0 generation even though they were not themselves exposed to overnutrition (F1 generation). In the second generation of offspring (F2 generation), fasting hyperglycemia and glucose intolerance were still apparent by 4 months of age [66]. In mouse as in nonhuman primates, in addition to impairing adiposity, maternal high-fat diet induces in the offspring an increase in gluconeogenic enzyme expression [67] or a reduction in liver insulin signalling and activation of JNK and IKK beta [68]. These studies thus provide a mechanism of action for the fatty liver and insulin resistance observed in those offspring. In line with the previous observations, offspring born to dams fed a fat-rich diet throughout gestation and lactation had a dysfunctional adipose tissue as characterized by impaired adipokines production (leptin, adiponectin) and enzymatic function (lipoprotein lipase and hormone-sensitive lipase). These defaults were worsened when the offspring were themselves fed the fat-rich diet after weaning [69]. Thus, *in utero* exposure to maternal obesity and unhealthy diet pattern *per se* can program multiple aspects of energy-balance regulation in the offspring that may be the leading cause of perpetuated adult metabolic diseases.

#### 5.2.2. The Epigenetic Matter

One possible mechanism by which obesity and metabolic disturbances can occur in offspring is related to epigenetic modifications of genes which may be induced by the *in utero* environment and result in gene expression without altering DNA sequences. Epigenetic changes are defined as the transmission of DNA or RNA activity without modifying the nucleotide sequence. Epigenetic misprogramming during development by maternal nutrition or by obesity-related metabolic milieu is now widely thought to have a persistent effect on the developmental plasticity of the foetus leading to obesity-related diseases in children [70]. Studies suggest that maternal obesity, via a pro-inflammatory milieu, insulin resistance, or other hormonal factors, causes epigenetic programming of the foetus [3, 71] which predisposes to the development of obesity and type 2 diabetes mellitus early in life, thus perpetuating the vicious circle of obesity. 

Recently, Plagemann and colleagues reported that neonatal overfeeding by culling pups to smaller litters size during lactation resulted in early weight gain, obesity, hyperleptinemia, hyperglycemia, increased insulin to glucose ratio and increased insulin receptor promoter (IRP) methylation in the hypothalamus [72]. Moreover, by performing longitudinal analysis of the data, blood glucose was positively associated to IRP methylation (*r* = 0.52; *P* = 0.04), independently of group (overfed or control). These data are in accordance with the observation that one mechanism by which glucose intolerance could occur in rat offspring exposed to high fat diet may be through increased hepatic gluconeogenesis and histone modification of the offspring liver Pkc1 gene which encodes the phosphoenolpyruvate carboxykinase 1 enzyme [73]. These data thus suggest that obese women carry a “milieu” that can potentially impair the developing child epigenomic-associated metabolic status and then lead to adulthood diseases.

#### 5.2.3. The Placental Effect

Another possible mechanism by which maternal nutrition can impact on the offspring may be through placental effects. Recently, a prospective observational study using a nonhuman primate model gave important insight into the consequences of high-fat diet-induced maternal obesity. In comparison to control mothers and independently of their BMI status, those mothers receiving the high-fat diet had a reduction in uterine blood flow and increase placental inflammation. Furthermore, mothers on the high-fat diet who developed obesity and displayed signs of hyperinsulinemia demonstrated a further decrease in their placental blood flow on the fetal side, thus favouring placental ischemia and stillbirth [74]. Moreover, when female C57BL/6 mice were fed a high-fat diet for 1 month prior to conception and throughout gestation, in addition to developing obesity and insulin resistance, their placentas lacked trophoblast cells and showed increased signs of oxidative stress, as compared to controls [75]. 

In human pregnancies complicated by obesity, higher placental pro-inflammatory cytokines and higher circulating IL-6 were measured [76]. Accordingly, it was recently demonstrated that exposition of human primary trophoblastic cell culture to IL-6 stimulated fatty acid accumulation. The authors also showed that irrespective of BMI, maternal 3rd-trimester circulating IL-6 levels negatively correlates with placental lipoprotein lipase enzyme activity thereby decreasing fatty acid uptake by the placenta. However, these last results were not replicated in culture conditions [77]. Moreover, fetal adiposity was positively correlated to maternal IL-6 [78], and adult offspring born to IL-6-deficient dams were heavier, had increased adiposity, decreased insulin sensitivity and leptin concentration [79]. IL-6-deficient mice presented reduced leptin concentration in their milk and wild type cross-fostered to IL-6-deficient mice had increased weight and adipocyte size and showed altered hypothalamic gene expression. It can thus be speculated that placental inflammation generated by maternal obesity can contribute to the metabolic disturbance observed in the offspring. Collectively, these data associates maternal obesity to fetal programming of metabolic disease by impaired placental function.

## 6. Long-Term Health Effect of Gestational Diabetes on the Offspring

### 6.1. Clinical Studies

In addition to maternal obesity, prepregnancy diabetes or GDM has been associated with increased risk in the offspring of developing later in life obesity, insulin resistance, and type 2 diabetes [80, 81]. In a large prospective analysis including mother-infant pairs who were enrolled in the National Collaborative Perinatal Project, infants born from GDM mothers were more likely to have elevated birth weight and presented a higher BMI Z-score at 7 years of age [82]. In order to further deepen the mechanism by which GDM induces adiposity in children, a study tested 41 children born from mothers with GDM, 41 children born from diabetic fathers (and mother with normal glucose tolerance), and 548 children born from parents with both normal glucose tolerance (control) [83, 84]. In comparison to control newborns, those exposed to maternal diabetes were bigger while those born to diabetic fathers were lighter. Following up the children at 5 years of age, daughters of diabetic mothers developed larger subscapular and triceps skinfold thicknesses and displayed higher insulin concentrations during the oral glucose tolerance test (OGTT), which was accompanied by higher insulin increment. These daughters were also more prone to develop impaired glucose tolerance in comparison to control children (18.3% versus 1.7%). At 5 years of age, children of diabetic fathers had similar anthropometric data to controls, but daughters had an increased prevalence of impaired glucose tolerance (10.5% versus 1.7%). This data lead the authors to speculate that in children born to diabetic father, glucose intolerance in later life may be programmed by low birth weight. However, since daughters of diabetic mothers were characterized both by increased adiposity and lower insulin sensitivity, which was not the case for daughters of diabetic fathers, these findings pinpoint to the direct influence of the intrauterine milieu on fetal programming of later disease.

Regarding maternal glucose tolerance during pregnancy, as determined by the 2nd-trimester OGTT, it was found that the higher the maternal plasma glucose was increased during the test, the higher the cord plasma glucose to insulin ratio was decreased in comparison to cord blood of foetuses born to normoglycemic mothers, but with pro-insulin-to-insulin ratio maintained stable [85]. These results suggest that maternal diabetes affect fetal insulin sensibility rather than **β**-cell function and that this may lead to altered metabolic function in the children later in life. Accordingly, in a cohort of 21 children aged between 5 and 10 years, Bush and colleagues showed that 2nd-trimester glycemic level 1 hour after a 50 g oral glucose load was inversely correlated with insulin sensitivity assessment during a liquid meal tolerance test in the children, but positively associated with static **β**-cell response and this was independently of adiposity [86]. Taken together, these studies suggest that at a young age, children who were exposed to high glucose concentrations during their fetal life develop metabolic dysfunctions with time. Studies using gold standard techniques for metabolic assessment and larger cohort are needed to really conclude on the specific effects of high maternal glucose on the reprogramming of fetal functions.

### 6.2. Potential Mechanisms for the Long-Term Impacts of Gestational Diabetes on the Offspring

Animal studies were also conducted in order to pinpoint the influence of GDM on the transgenerational transmission of obesity and diabetes. In fact, already in the early 90s', studies performed in rodents showed that maternal hyperglycemia leads to overweight, impaired glucose tolerance, hyperinsulinemia and insulin resistance in the offspring later in life [87, 88]. Such metabolic consequences persisted through the F1 and F2 generations [87–89]. In mice, it was recently reported that these defects were perpetuated to the next generations through maternal and/or paternal lineages imprinted genes [90, 91]. Moreover, Bouchard and colleagues recently demonstrated, in placentas of mothers experiencing glucose intolerance during pregnancy, an inverse relationship between maternal plasma glucose concentration 2 hours after an OGTT and placental fetal side leptin DNA methylation. Increased placental leptin DNA methylation was also associated to lower cord blood leptin mRNA [92]. These results thus indicate that, as shown for maternal obesity, gestational hyperglycemia induces during fetal life an epigenetic mode of inheritance for the transmission of adulthood obesity and associated metabolic disturbances.

## 7. Interventional Studies Aimed at Reducing the Impact of Maternal Obesity and/or Gestational Diabetes on the Offspring

The question next arising is whether maternal weight loss (before or during pregnancy) can reverse or at least reduce the potential burden of obesity on the offspring. Or more generally, in those mothers who are overweight or obese and/or develop GDM, how can we reduce the risk of transgenerational transmission of obesity and diabetes to the developing child? 

In a population of glucose-intolerant subjects, the Diabetes Prevention Program (DPP) and the Finnish Diabetes Prevention Study (DPS) demonstrated the effectiveness of an intensive program of lifestyle modification, which was accompanied by modest weight loss, in order to prevent almost 60% of new cases of type 2 diabetes mellitus [93, 94]. Moreover, the American Dietetic Association [95], the American Society for Nutrition [95], and the British Fertility Society [96] have all recommended that overweight/obese women should be provided with assistance to lose weight prior to conception and maintain a healthy lifestyle to prevent excess weight gain during pregnancy. Furthermore, a recent review of the literature proposed that improved weight management by adequate nutritional diet may be successful in preventing GDM in pregnant women [97]. 

### 7.1. Lessons from Bariatric Surgery

Consistent with the importance for weight loss, most bariatric surgery has shown some beneficial [98, 99] effects on pregnancy outcomes in obese mothers and their neonates even though it was not the case in some other studies (as reviewed in [100]). Of note, bariatric surgery is also likely to affect patients' eating habits that contribute to improve obstetrical and neonatal complications in addition to weight loss. One lesson that we learned from bariatric surgery is that children born to mothers after weight loss due to bariatric surgery had a reduction in macrosomic prevalence in comparison to the siblings born before surgery, without difference in prevalence of low birth weight [101]. In the children born from a pregnancy occurring after the mother underwent bariatric surgery, their risk of developing obesity into adulthood was greatly reduced [101]. Moreover, in a retrospective study, it was shown that in women who delivered after a bariatric surgery, the incidence of GDM and caesarean was less than in pregnancies that occurred before surgery [102]. These data thus imply that maternal weight loss and improvement of metabolic state before pregnancy improve obstetrical and neonatal complications, as well as adulthood-programmed disease in the offspring.

### 7.2. Lifestyle Management and Weight Control before or during Pregnancy

In the next section, we will present clinical (summarized in Table 2) and animal (summarized in Table 3) studies that have examined the main interventions tested to reduce the impact of maternal obesity and gestational diabetes and how they can break the vicious circle that perpetuates the transmission of obesity and metabolic conditions to the next generations.

#### 7.2.1. Clinical Studies

Regarding lifestyle management during pregnancy, few studies assessing the role of a dietitan intervention alone have been published. A randomized controlled trial undertaken in Belgium enrolled 122 obese pregnant women in 3 groups receiving either nutritional advice from a brochure; the brochure and lifestyle education by a dietitian; or no intervention [103]. By assessing dietitian intervention using food log book or questionnaires, the authors found that while their intervention positively impacted on nutritional habits of participants, it did not result in any improvement of obstetrical or neonatal outcomes. On the other hand, Wolff et al. showed that when obese pregnant women were assisting to frequent nutritional consultation with a specialized dietitian (ten 1-hour sessions during pregnancy), they gained less gestational weight and reduced their fasting insulin, glucose and leptin concentrations, thus suggesting improved metabolic control [104]. However, as in the previous paper, similar obstetrical and neonatal complications between intervention and control women were observed. These studies thus suggest that in order to achieve more optimal weight gain during pregnancy with a nutritional approach alone, it seems important that the participant benefits from individualized professional advices.

The results of studies assessing the benefits of exercise alone either before and/or during pregnancy are worth mentioning. In a small cohort of obese pregnant women, Callaway and colleagues showed that even though an increase in energy expenditure of 900 kcal/week was achieved by 28 weeks of gestation with an individualized exercise program, HOMA-IR and GDM outcomes were not different in comparison to the group receiving usual care, and the authors concluded that the efficiency of their intervention was not convincing [105]. Nevertheless, they showed that by 28 weeks, fasting glucose was significantly reduced and that by 36 weeks, fasting insulin was significantly reduced by 28% in mothers of the intervention group, thus suggesting that these metabolic gains still may show some benefits on a longer-term basis in their children. Clapp et al. also found that in women physically active before pregnancy, those who sustained intensive activity throughout pregnancy gain less gestational weight than those who stopped physical activity at the beginning of pregnancy [106]. Moreover, they showed beneficial effects on the newborns such as reduced birth weight, ponderal index, percent body fat and fat mass. Moreover, these benefits were maintained at 5 years of age as these children had reduced weight, ponderal index and skinfolds as well as presented improved neurodevelopmental skills. A recent paper reviewing prospective, retrospective and cross-sectional studies assessing prepregnancy and early pregnancy physical activity in over 35.000 and 4.400 participants, respectively, suggested that these women were at reduced risk of GDM [118].

Other trials aimed to improve maternal, neonatal and long-term offspring outcomes by lifestyle management. Indeed, it was shown by Artal and colleagues that, in obese women with GDM, the addition of physical activity to improved dietary habits resulted in less gestational gain weight when compared to the effect of diet alone, without compromising neonatal outcomes [107]. Quinlivan and colleagues performed a randomized-controlled trial enrolling overweight or obese Australian pregnant participants allocated to standard care or a 4-step approach consisting of a visit with an interdisciplinary team including: (1) an obstetrician for continuity of maternal care, (2) a food technologist for nutritional habits and for providing food information, (3) a nurse performing weight measurements and (4) a psychologist to evaluate signs of depression and anxiety [108]. This interdisciplinary lifestyle intervention lead to a significant reduction in gestational weight gain (7.0 versus 13.8 kg) and in the incidence of GDM (6 versus 29%), as compared to standard care. In spite of these clinically significant differences in obstetrical outcomes, neonate weights were similar between the 2 groups. Similarly, a group of Finland's pregnant women at risk for GDM, based on the results of an OGTT performed during first trimester, were randomised to the intervention group, consisting of dietary and physical activity counselling, or in the control group [109]. Weight gain was slightly, although not significantly, reduced in the intervention group (11.4 versus 13.9; *P* = 0.06), but no difference was observed between groups regarding glucose tolerance during 2nd trimester. However, although obstetrical and neonatal outcomes were similar between groups, they showed a slight, but significant increase birth weight in the intervention versus close follow-up group. Other studies have shown that lifestyle improvement by nutritional or physical activity behavioural modifications during pregnancy resulted in recommended gestational weight gain or lower gain than with control intervention [110, 111]. Altogether, these trials and others (including review [122] and meta-analysis [123]) suggest that it is feasible to improve weight management and/or the metabolic state of obese or at-risk women during pregnancy, without negative impact on fetal outcomes. However, results are somewhat scarce, controversial and inconsistent regarding the impact on maternal and fetal medical complications, and long-term follow-up studies are lacking in order to determine whether they have long lasting metabolic benefits on the offspring. 

The benefits of controlling adequately maternal hyperglycemia during pregnancy are now well established and have influenced international guidelines on the diagnosis and treatment of gestational hyperglycaemic disorders. The Australian Carbohydrate Intolerance in Pregnant Women (ACHOIS) study showed that in women with mild impaired glucose tolerance but treated with dietary advices and if needed, insulin, macrosomia incidence and serious neonatal complications were significantly reduced as compared to the routine care group [112]. However, intervention was associated with higher admission to neonatal care unit and increased rate of labor induction although caesarean rate were similar. This intervention was not associated with any change in BMI in children at 4-5 years of age between groups [113]. In a randomized controlled trial held in the US, Landon and colleagues showed similar results in women with mild gestational diabetes enrolled in the intervention group consisting of a dietary intervention, self-monitoring of blood glucose and, if needed, insulin therapy [114]. In comparison to control group who received usual care, the intervention did not result in improved neonatal complications as in the ACHOIS study, but it resulted in better fetal growth, as shown by reduced incidence of macrosomia, large-for-gestational age as well as fetal fat mass [114]. Unlike the ACHOIS study, this trial found a reduction in preeclampsia, caesarean and shoulder dystocia. Results of the long-term follow-up of the offspring are not yet available. However, in 71 children assessed between 7–11 years of age, Malcolm and colleagues found that interventions in women with gestational diabetes aiming for either minimal or tight glycemic control during pregnancy [115] were equally effective for the prevention of impaired glucose tolerance [116]. However, this conclusion needs to be interpreted with caution because no control group was included and this study was limited by its relatively small sample size. 

Since carbohydrates are the main determinant of postprandial blood glucose level [124], intervention trials were assessed or are presently being held to establish the effect of low glycemic index diet during pregnancy on neonatal outcomes [125, 126]. Accordingly, one interventional study conducted in healthy pregnant women proposed that low glycemic index diet reduces the incidence of macrosomia [117]. In pregnancies complicated by GDM, it is generally accepted that a low glycemic index diet is indicated because it was shown to facilitate glucose control without compromising obstetric and fetal outcomes [127, 128]. In a recent systematic review of literature on low glycemic index diet, Louie et al. proposed that until results of larger randomised control trials are available, low glycemic index diet should not be introduced into clinical practice, except for mothers with GDM, because data are not consistent and supported by large cohorts [129]. 

In brief, these clinical intervention trials showed some benefits in terms of improved gestational weight gain but few provided convincing data for better impact on the neonates. However, limitations include mainly small number of participants (lack of power) and possible publication bias. As it is otherwise satisfactory to observe that these interventions were not detrimental to the fetus, the next question arising is whether they have a long lasting impact on later childhood obesity or metabolic function.

#### 7.2.2. Animal Studies

Although the impact of gestational obesity and diabetes on the offspring is well described, human trials conducted to determine whether maternal interventions could break down the vicious cycle on future generation are still at their infancy. Therefore, only animal studies can help us to make some inferences for the moment. In fact, the potential beneficial impact of nutritional intervention were reported in the yellow Agouti mice and in rats born to fat-fed mothers. In the yellow Agouti mice, authors showed that *in utero* exposure to the dietary antioxidant genistein, at levels present in human adult populations consuming high-soy diets, reduces obesity by altering the epigenome in those mice [119]. In addition, offspring born from dams fed a fat Westernized diet developed by 2 weeks of age greater adiposity and glucose intolerance than those born from dams fed the control diet [120]. However, offspring born from dams fed the Westernized diet and supplemented with antioxidant normalized their fat mass as well as their leptin, glucose, insulin and non-esterified fatty acid blood concentrations to levels similar to the control group.

Zambrano and colleague showed that giving a normal chow to obese female rats 1 months prior to mating and throughout pregnancy and lactation in comparison to obese rats who were kept on the obesogenic diet throughout entire study period resulted in dams with lower gestational weight gain but in offspring of similar phenotype and weight from birth to 150 days of age [121]. These results are in agreement with the previously reported literature in human where maternal interventions with the aim to reduce gestational weight gain had no effect on the foetuses birth weight. However, by 21 days of age, offspring born to mothers on the intervention arm significantly improved their high fat mass, hypertriglyceridemia, hyperleptinemia, hyperinsulinemia and insulin resistance, in comparison to rats born from untreated obese dams, and almost to the same levels as in rats born from non-obese dams. By 150 days of age, improvements in leptinemia, fat mass and adipocyte cell size were still observable [121]. 

These results suggest that maternal intervention aimed at reducing gestational weight gain has tremendous long lasting metabolic effects on the offspring, even if fetal and neonatal profile were similar or slightly changed. It is therefore possible to extrapolate that even though interventions in humans did not result in improved newborn phenotype, beneficial effects on the intrauterine milieu may have long lasting effect on adulthood health of the offspring.

## 8. Conclusion

In summary, obesity is a common and growing condition affecting women health. Accordingly, women are more likely to enter pregnancy being overweight and often exceed the recommended gestational weight gain. As one major weight-associated complication, GDM may lead to profound and long lasting effect in the child. Moreover, even without the development of GDM, gestational maternal weight increases the future risk of cardiometabolic conditions in the offspring. Fetal programming of metabolic function induced by obesity and GDM may have intergenerational effect and thus, perpetuate the burden of such conditions. Mechanisms by which reprogramming of fetal function might occur is directly through maternal metabolic and hormonal effects, epigenetic alterations or impaired placental function. Periconceptional weight loss interventions have demonstrated their ability to reverse the impacts of maternal obesity and GDM on the child and are of great importance for the prevention of future cardiometabolic risks in the offspring, and may thus be the best approach to break the vicious circle of intergenerational propagation of obesity and diabetes. However, the nature and the timing of intervention should be carefully considered because it could also by itself induce organ reprogramming and potential long-term effect on the offspring [130, 131]. In addition, larger cohorts and long-term randomized controlled trials are necessary to provide robust conclusions.

## Figures and Tables

**Table 1:** Recommendation given by the Institute of Medicine in 2009 for appropriate gestational weight gain based on preconception body mass index (BMI).

Preconception BMI (kg/m^2^)	Total weight gain during pregnancy	Weight gain during 2nd and 3rd trimesters (per week)
<18.5	28–40 lbs/13–18 kg	1.0–1.3 lbs/0.5-0.6 kg
18.5–24.9	25–35 lbs/11–16 kg	0.8–1.0 lbs/0.4-0.5 kg
25.0–29.9	15–25 lbs/7–11 kg	0.5–0.7 lbs/0.2-0.3 kg
≥30.0	11–20 lbs/5–9 kg	0.4–0.6 lbs/0.2-0.3 kg

Adapted from [4].

**Table 2:** Most important human clinical interventions trials performed in pregnant mothers to reduce or improve maternal, neonatal, and child outcomes.

Study	Population	Study design	Intervention	Maternal outcomes	Gestational or obstetrical outcomes	Neonatal outcomes	Long-term offspring outcomes
Guelinckx et al. 2010*Am J Clin Nutr* [103]	*n* = 122, nondiabetic obese (BMI > 29) pregnant (<15 weeks) women	Randomized controlled trial	*Passive group*: brochure on general advices at 1st prenatal visit *Active group*: brochure + 3 group sessions with dietitian during pregnancy on nutrition and physical activity *Control group*: routine care	*Gestational weight gain*: similar *GDM incidence*: N/A *Intervention impact*: in active group, improved nutritional habits during pregnancy and compared to other groups; similar physical activity	*Intervention impact*: similar	*Intervention impact*: similar birth weight	N/A

Wolff et al., 2008 *Int J Obes* [104]	*n* = 50, nondiabetic obese (BMI = 35) pregnant (<15 weeks) women	Randomized controlled trial	*Intervention group*: ten individual sessions with dietitian during pregnancy to improve gestational weight gain with energy intake restriction *Control group*: routine care	*Gestational weight gain*: ↓ 6.7 kg versus controls (*P* = 0.002) *GDM incidence*: similar *Intervention impact*: at 27 weeks: ↓insulin, ↓leptin versus controls at 36 weeks: ↓ insulin, ↓ glucose versus controls	*Intervention impact*: similar	*Intervention impact*: similar birth weight, placental weight, head, and abdominal circumference	N/A

Callaway et al., 2010 *Diabetes Care* [105]	*n* = 50, obese (BMI ≥ 30) pregnant women *participants with type 1 diabetes excluded	Randomized controlled trial	*Intervention group*: individual exercise program with energy expenditure (EE) goal of 900 kcal/week from 12-week gestation to delivery *Control group*: routine care	*Gestational weight gain*: N/A *GDM incidence*: in intervention group, 12% ↑ at 12 week (*P* = 0.07) and similar at 28 week (*P* = 0.57) *Intervention impact*: at 28 weeks: ↑ EE (*P* = 0.04), ↓ fasting glucose (*P* = 0.03), at 36 weeks: ↓ fasting insulin (*P* = 0.05)	N/A	N/A	N/A

Clapp. 1996*J Pediatr* [106]	*n* = 40, recruited before pregnancy, healthy (BMI not mentioned) caucasian women. Offspring aged 5 yrs: *n* = 12 girls and 8 boys in each group	Prospective cohort study	*Active women*: healthy and physically active before and throughout pregnancy. *Control women*: healthy and physically active before and stop at initiation of pregnancy No intervention in children	*Gestational weight gain*: ↓ in active versus control group *GDM incidence*: N/A	N/A	*Intervention impact*: ↓ birth weight, ponderal index, fat mass, body fat %, and abdominal circumference (*P* < 0.01)	*Intervention impact at 5 years of age*: ↓ weight, skinfolds, arm area fat mass (*P* < 0.01), and ponderal index (*P* < 0.05); ↑general intelligence, language skill (*P* < 0.01), and neurodevelopmental score (*P* < 0.05)

Artal et al., 2007 *Appl Physiol Nutr Metab* [107]	*n* = 96, overweight or obese (BMI > 25) pregnant (<33 weeks) women with GDM (no insulin)	Randomized controlled trial No control group without intervention	*Diet (D) group*: individual counseling on diet and weight gain goal according to BMI *Exercise and diet (ED) group*: same plus individual counseling on moderate exercise All received usual GDM management	*Gestational weight gain*: ↓ in ED versus D group (*P* < 0.05) *GDM incidence*: 1st trimester: similar 2nd trimester: similar in term of prescription related to insulin to maintain glucose.	*Intervention impact*: similar caesarean rates	*Intervention impact*: similar for macrosomia, small for gestational age and birth weight	N/A

Quinlivan et al., 2011*Aust N Z J Obstet Gynaecol* [108]	*n* = 124, overweight or obese (BMI > 25) pregnant women	Randomized controlled trial	*Intervention group*: 4 steps individual approach: (1) continuity of care provider, (2) weighting at each visit, (3) brief dietary intervention by a food technologist, and (4) psychological support *Control group*: routine care	*Gestational weight gain*: ↓ in intervention versus control (*P* < 0.001) *GDM incidence*: ↓ in intervention versus control (*P* < 0.04)	*Intervention impact*: N/A	*Intervention impact*: similar birth weight	N/A

Korpi-Hyovalti et al., 2011 *BMC Public health* [109]	*n* = 54, high risk for GDM pregnant (8–12 weeks) women irrespective of BMI at inclusion	Open multicenter randomized controlled trial	*Intervention group*: individual counseling on nutrition (dietitian) and physical activity (physiotherapist) *Control group*: close followup	*Gestational weight gain*: small ↓ in intervention group (*P* = 0.062) *GDM incidence*: similar	*Intervention impact*: similar	*Intervention impact*: heavier birth weight (*P* = 0.047); similar rates of macrosomia, admissions to NICU and respiratory distress	N/A

Lindholm et al., 2010 *Acta Obstet Gynecol Scand* [110]	*n* = 25, overweight (BMI 30–35, *n* = 11) or obese (BMI > 35, *n* = 14) pregnant women	Uncontrolled prospective intervention study	Individual counseling with midwife every 2 weeks + 2 group sessions from 1st trimester to delivery on diet and exercise. One initial consultation with dietitian Goal: gestational weight gain ≤ 6 kg	*Gestational weight gain*: 14/25 reached goals; ↓ in prepregnancy BMI > 35 versus 30–35 (*P* = 0.001) *GDM incidence*: as expected	*Intervention impact*: in prepregnancy BMI > 35 versus 30–35:↑ gestational weeks (*P* = 0.04), ↓ caesarean (*P* = 0.04)	*Intervention impact*: fetal growth and birth weight: as expected	N/A

Shirazian et al., 2010 *Am J Perinatol* [111]	*n* = 41, nondiabetic obese (BMI > 30) pregnant women	Prospective study with historical controls	*Intervention group*: individual counseling on healthy diet and exercise, ≥ 5 one-on-one counselling or phone calls + ≥ 1 seminar/trimester x/trimester. Weight gain goal ≤ 15 lbs. *Control group*: routine care	*Gestational weight gain*:1/2 of control (*P* = 0.003) *GDM incidence*: similar	*Intervention impact*: similar	*Intervention impact*: similar birth weight and rates of fetal complications	N/A

Crowther et al., 2005 *N Engl J Med* [112] Gillman et al., 2010 *Diabetes Care* [113]	*n* = 1 000, mild GDM pregnant women (median BMI = 26) Offspring aged 4-5 yrs: *n* = 94 (intervention group); *n* = 105 (control group)	Multicenter randomized controlled trial ACHOIS study	*Intervention group*: individual dietary and lifestyle counseling, usual GDM care, insulin therapy in 20% of women; from recruitment (24–34 week gestation) to delivery *Control group*: routine care No intervention in children	*Gestational weight gain*: ↓ in intervention versus control (*P* = 0.01) *GDM incidence*: similar *Intervention impact*: ↑ postpartum quality of life	*Intervention impact*: ↑ induction of labor (*P* < 0.001), similar caesarean rates	*Intervention impact*: ↓ perinatal complications (*P* = 0.01), ↑ admission to neonatal nursery (*P* = 0.01), ↓ birth weight (*P* < 0.001), ↓ macrosomia (<0.001)	*Intervention impact*: in offspring 4-5 y.o., similar BMI *Z*-score, and proportion of BMI ≥ 85th percentile

Landon et al., 2009 *N Engl J Med* [114]	*n* = 900, mild GDM pregnant women (median BMI = 30)	Multicenter randomized controlled trial	*Intervention group*: individual nutritional counseling, usual GDM care, insulin therapy (required in 37 women); from recruitment (24–31 weeks) to delivery *Control group*: routine care, insulin therapy (required in 2 women)	*Gestational weight gain*: ↓ in intervention versus control (*P* < 0.001). *GDM incidence*: N/A *Intervention impact*: achievement of maternal glucose targets	*Intervention impact*: ↓ caesarean (*P* = 0.01), shoulder dystocia (*P* = 0.02), preeclampsia, or hypertension (*P* = 0.01)	*Intervention impact*: ↓ birth weight (*P* < 0.001), macrosomia (*P* < 0.001), fat mass (*P* = 0.003); similar perinatal complications, small for gestational age, admissions to NICU, and respiratory distress	N/A

Garner et al., 1997 * Am J Obstet Gynecol* [115] And Malcolm et al., 2006 *Diabet Med* [116]	*n* = 300, low-risk pregnant (24–32 weeks) women with GDM Offspring aged 7–11 yrs: *n* = 46 (intervention group), *n* = 25 (control group)	Randomized controlled trial	*Intervention group*: individual dietary counseling by dietitian biweekly, usual GDM care, insulin therapy (required in 36 women) *Control group*: not seen by dietitian; recommended to eat unrestrictedly according to Canada food guide; biweekly self-glucose monitoring No intervention in children	*Gestational weight gain*: similar *GDM incidence*: similar OGTT area under the curves *Intervention impact*: at 28–30 weeks: ↑ preprandial glucose levels (*P* = 0.001) at 36–38 weeks: ↓ preprandial (*P* = 0.035) and 1-hour postprandial (*P* = 0.009) glucose levels	*Intervention impact*: similar caesarean rates	*Intervention impact*: similar birth weight, perinatal complications	*Intervention impact*: similar mean fasting glucose, mean fasting insulin, and mean 2 hrs glucose and insulin; 5 children born to mothers from intervention group had AGT

Moses et al., 2006 * Am J Clin Nutr* [117]	*n* = 70, pregnant (12–16 weeks) women *Participants with conditions associated to abnormal glucose metabolism and insulin resistance problem were excluded	Randomized controlled trial No control group without intervention	*Low-glycemic index group*: individual nutritional counseling by dietitian 5 times during pregnancy on recommended nutritional intake plus low-glycemic index diet *High-glycemic index group*: idem except for recommendation of high- glycemic index diet	*Gestational weight gain*: similar *GDM incidence*: similar (only 1 case) *Intervention impact*: in low versus high-glycemic index group: ↓ glycemic index (*P* < 0.001), fasting glucose (*P* = 0.034)Within low-glycemic index group: ↓ fasting glucose (*P* = 0.001)	*Intervention impact*: in low- versus high-glycemic index group: similar caesarean rates	*Intervention impact*: in low- versus high-glycemic index group: ↓birth weight (*P* = 0.051), macrosomia (*P* = 0.001), ponderal index (*P* = 0.03); similar perinatal complications, small for gestational age, admissions to NICU, respiratory distress	N/A

ACHOIS: Australian Carbohydrate Intolerance in Pregnant Women study, AGT: abnormal glucose tolerance, BMI: body mass index, GDM: gestational diabetes mellitus, MET: metabolic equivalent task, NICU: neonatal intensive care unit, N/A: not applicable, OGTT: oral glucose tolerance test.

**Table 3:** Most important interventions performed in pregnant animals to reduce or improve maternal, neonatal, and offspring outcomes.

Study	Animal model	Intervention	Maternal outcomes	Gestational or obstetrical outcomes	Neonatal outcomes	Long-term offspring outcome
Dolinoy et al., 2006 *Environ Health Perspect* [119]	Heterozygous viable yellow agouti (A^vy^) mice *n* = 15, unsupplemented litters (52 offspring) *n* = 12, genistein supplemented (44 offspring)	Females (8–10 weeks of age) received phytoestrogen-free modified AIN-93G diet or modified AIN-93G diet supplemented with 250 mg/kg of genistein (comparable to human high-soy diet) Diet provided 2 weeks before mating, throughout pregnancy and lactation	N/A	N/A	*Day 21*:similar litter size, wean weight, percent survival, sex ratio; maternal supplementation favored pseudoagouti versus full agouti (yellow coat) phenotype (*P* = 0.0005) + ↑ Agouti gene methylation	Agouti gene methylation highly correlated between day 21 and 150 in offspring whose mothers received supplement At week 60: ↓ weight in pseudoagouti versus full agouti mice (*P* = 0.0001) Pseudoagouti: normal weight versus full agouti (more obese)

Sen et al., 2010 *Diabetes* [120]	Female Sprague-Dawley rats *Diets*: control (C, *n* = 10); control + antioxidant (CAox, *n* = 10); Western (W, *n* = 10), high protein + fat (HP + F, *n* = 10); W + Aox (WAox, *n* = 10)	Female rats received diets from age 4 to 13–15 weeks (end of gestation). Litters culled to 8 pups. Studies performed in male offspring only. At weaning, offered control diet.	*End of gestation*:↑ gestational weight gain in dams offered W and WAox versus C; similar glucose and food consumption;↑ insulin, free-fatty acid (FFA) and leptin in W versus C, but ↓ in WAox versus W	N/A	*Embryos*:in W versus C: ↑oxidative stress, ↓ in WAox *Birth*: similar weight In W versus C: ↑ FFA, insulin and leptin *2 weeks*: in W versus C: ↑ fat mass, FFA, insulin, leptin, proadipogenic, and lipogenic genes expression, but↓ in WAox versus W	*2 months*: in W versus C:↑ total and central fat mass, insulin, leptin, proadipogenic and lipogenic genes expression, but↓ in WAox versus W; ↑ impaired glucose tolerance, but normalized in WAox (similar to C)

Zambrano, et al. 2010 * J Physiol* [121]	Female albino Wistar rats *n* = 5; control *n* = 5; obesogenic; *n* = 5 dietary intervention (DINT)	At 3 weeks, females rats received obesogenic (0; *n* = 10) or control (C; *n* = 5) diets At 90 days, O dams were either continued on O diet (*n* = 5) or receiving the C diet (DINT; *n* = 5) One month after, animals were bred and continued prepregnancy diet throughout pregnancy and lactation Litters culled to 10 pups; pup sex ratio 1 : 1	N/A	*90 days*:in O and DINT versus C: ↑weight *Breeding and * *delivery*: in O versus C: ↑weight, but ↓ in DINT versus O *Weaning*: in O versus C: ↑leptin, but ↓ in DINT versus O	*Birth and weaning*: similar morphometric variable *Weaning*: in O versus C: ↑subcutaneous fat tissue, serum triglycerides, leptin, insulin, but ↓ in DINT versus O; similar glucose despite ↑ insulin (suggesting insulin resistance), but ↓ in DINT versus O	*Postnatal day 120*: in O versus C: ↑serum glucose, insulin, insulin resistance, but similar glucose with ↑ insulin in DINT versus C (suggesting partial recovery of insulin resistance) *Postnatal day 150*: similar body weight In O versus C: ↑ fat and leptin, larger fat cell size In DINT versus C: non-significant ↓ in fat mass and fat cell size
